# Secondary Worsening Following DYT1 Dystonia Deep Brain Stimulation: A Multi-country Cohort

**DOI:** 10.3389/fnhum.2020.00242

**Published:** 2020-06-25

**Authors:** Takashi Tsuboi, Laura Cif, Philippe Coubes, Jill L. Ostrem, Danilo A. Romero, Yasushi Miyagi, Andres M. Lozano, Philippe De Vloo, Ihtsham Haq, Fangang Meng, Nutan Sharma, Laurie J. Ozelius, Aparna Wagle Shukla, James H. Cauraugh, Kelly D. Foote, Michael S. Okun

**Affiliations:** ^1^Department of Neurology, Norman Fixel Institute for Neurological Diseases, University of Florida, Gainesville, FL, United States; ^2^Department of Neurology, Nagoya University Graduate School of Medicine, Nagoya, Japan; ^3^Department of Neurology, University Hospital Montpellier, Montpellier, France; ^4^Department of Neurosurgery, University Hospital Montpellier, Montpellier, France; ^5^Department of Neurology, University of California, San Francisco, San Francisco, CA, United States; ^6^Department of Stereotactic and Functional Neurosurgery, Fukuoka Mirai Hospital, Fukuoka, Japan; ^7^Division of Neurosurgery, Toronto Western Hospital Krembil Neuroscience Center, Toronto, ON, Canada; ^8^Department of Neurosurgery, University of Toronto, Toronto, ON, Canada; ^9^Department of Neurosurgery, KU Leuven, Leuven, Belgium; ^10^Department of Neurology, Wake Forest School of Medicine, Winston-Salem, NC, United States; ^11^Beijing Neurosurgical Institute, Beijing Tiantan Hospital, Capital Medical University, Beijing, China; ^12^Department of Neurology, Massachusetts General Hospital, Charlestown, MA, United States; ^13^Department of Applied Physiology and Kinesiology, University of Florida, Gainesville, FL, United States; ^14^Department of Neurosurgery, Norman Fixel Institute for Neurological Diseases, University of Florida, Gainesville, FL, United States

**Keywords:** DYT1, dystonia, deep brain stimulation, globus pallidus internus, pallidum

## Abstract

**Objective**: To reveal clinical characteristics of suboptimal responses to deep brain stimulation (DBS) in a multi-country DYT1 dystonia cohort.

**Methods**: In this multi-country multi-center retrospective study, we analyzed the clinical data of DYT1 patients who experienced suboptimal responses to DBS defined as <30% improvement in dystonia scales at the last follow-up compared with baseline. We used a literature-driven historical cohort of 112 DYT1 patients for comparison.

**Results**: Approximately 8% of our study cohort (11 out of 132) experienced suboptimal responses to DBS. Compared with the historical cohort, the multi-country cohort with suboptimal responses had a significantly younger age at onset (mean, 7.0 vs. 8.4 years; *p* = 0.025) and younger age at DBS (mean, 12.0 vs. 18.6 years; *p* = 0.019). Additionally, cranial involvement was more common in the multi-country cohort (before DBS, 64% vs. 45%, *p* = 0.074; before or after DBS, 91% vs. 47%, *p* = 0.001). Mean motor improvement at the last follow-up from baseline were 0% and 66% for the multi-country and historical cohorts, respectively. All 11 patients of the multi-country cohort had generalization of dystonia within 2.5 years after disease onset. All patients experienced dystonia improvement of >30% postoperatively; however, secondary worsening of dystonia commenced between 6 months and 3 years following DBS. The improvement at the last follow-up was less than 30% despite optimally-placed leads, a trial of multiple programming settings, and additional DBS surgeries in all patients. The on-/off-stimulation comparison at the long-term follow-up demonstrated beneficial effects of DBS despite missing the threshold of 30% improvement over baseline.

**Conclusion**: Approximately 8% of patients represent a more aggressive phenotype of DYT1 dystonia characterized by younger age at onset, faster disease progression, and cranial involvement, which seems to be associated with long-term suboptimal responses to DBS (e.g., secondary worsening). This information could be useful for both clinicians and patients in clinical decision making and patient counseling before and following DBS implantations. Patients with this phenotype may have different neuroplasticity, neurogenetics, or possibly distinct neurophysiology.

## Introduction

DYT1 (DYT-*TOR1A*) is the most common cause of inherited isolated dystonia, and almost all patients possess the same mutation in the *TOR1A* gene (c.907_909delGAG; Charlesworth et al., 2013). Dystonia symptoms of DYT1 patients most often begin in an arm or leg during childhood or adolescence (Lee et al., 2012). Dystonia spreads to other body regions and becomes generalized over months to years in up to half of patients (Fasano et al., 2006; Lee et al., 2012). Cranial involvement is however uncommon.

Globus pallidus internus deep brain stimulation (GPi DBS) improves motor function and quality of life in dystonia patients and is considered a therapeutic option for patients with medically-refractory dystonia (Vidailhet et al., 2005; Volkmann et al., 2012; Tsuboi et al., 2019). In 2000, excellent DBS outcomes in patients with DYT1 were first reported by Coubes et al. (2000). Subsequently, retrospective studies and a meta-analysis suggested that *TOR1A* mutation-positive status was a clinical predictive factor for a better DBS outcome (Vasques et al., 2009; Andrews et al., 2010; Borggraefe et al., 2010; FitzGerald et al., 2014; Moro et al., 2017; Artusi et al., 2020). Additionally, in the largest cohort to date of patients with DYT1 dystonia who underwent GPi DBS (*n* = 47), improvement in dystonia severity by >80% was maintained up to 7 years after surgery (Panov et al., 2013). In contrast, there have been a minority of individual DYT1 cases with suboptimal responses to GPi DBS (Krause et al., 2004, 2016; Starr et al., 2006; Mehrkens et al., 2009; Cif et al., 2010; Markun et al., 2012; Miyagi and Koike, 2013; Ben-Haim et al., 2016; Pauls et al., 2017; Tsuboi et al., 2019). These less-than-expected responses in some cases could be attributable to suboptimal lead positions, non-optimized programming, or skeletal deformities. However, there are cases with suboptimal outcomes that remain unexplained, and there seem to be emerging cases of secondary worsening (Cif et al., 2010; Miyagi and Koike, 2013; Tsuboi et al., 2019). Because only a few cases of DYT1 dystonia are operated each year even in expert centers, the number of reported patients with suboptimal responses is limited. Consequently, the clinical characteristics of this cohort remain undefined.

We performed a multi-country multi-center retrospective study to investigate the characteristics of patients with DYT1 dystonia who experienced suboptimal responses to DBS. Additionally, we compared this unique cohort of patients to a historical cohort of DYT1 DBS patients to better define the potential characteristics which may differentiate responses.

## Materials and Methods

### Study Design

This retrospective study was approved by the University of Florida Institutional Review Board (IRB201900356), and each participating center had local IRB approval for the inclusion of their data in this study. We collected patient information that met the following inclusion criteria: (1) isolated dystonia; (2) a c.907_909delGAG mutation in the *TOR1A* gene; (3) bilateral GPi DBS performed at one of the participating expert centers; (4) no history of prior stereotactic brain surgery other than DBS; (5) preoperative clinical assessments with postoperative assessments at 1 year or longer; and (6) suboptimal responses to DBS (defined as <30% improvement in the Burke-Fahn-Marsden Dystonia Rating Scale motor score (BFMDRS-M) or the Unified Dystonia Rating Scale (UDRS) at the last follow-up compared with the baseline status (Burke et al., 1985; Comella et al., 2003). This 30% threshold was chosen arbitrarily as a clinically relevant response (Pauls et al., 2017). Earlier studies reported an excellent correlation between the BFMDRS-M and UDRS (0.977) and that percentage improvement after DBS in dystonia patients was similar between these scales (Comella et al., 2003; Susatia et al., 2010). Therefore, we considered percentage improvement based on these scales equivalent. The DBS programming data, the DBS lead positions, and the changes in motor scores, as well as detailed descriptive clinical information, were collected and analyzed.

### The Historical Cohort

To create a historical cohort of DYT1 patients treated with GPi DBS, a literature search was conducted using PubMed/Medline, Embase, and Cochrane databases in May 2019 with the following search terms: deep brain stimulation, neurostimulation, DBS, DYT1, and TOR1A. The search syntax is available in Supplementary Material. The reference lists for each of the identified articles were used to explore additional relevant publications. We considered both pure DYT1 cohorts and mixed cohorts including DYT1 patients for inclusion. The inclusion criteria were as follows: (1) DYT1 dystonia; (2) bilateral GPi DBS; (3) no history of prior stereotactic brain surgery other than DBS; and (4) preoperative clinical assessments with postoperative assessments at 1 year or longer. The minimum required information for individual patients was the age at onset, the age at DBS surgery, disease duration before DBS, and preoperative and postoperative motor scores according to either the BFMDRS-M or the UDRS. For articles in which only two of the following were provided (i.e., age at onset, age at DBS, and disease duration before DBS), the missing value was calculated using the other values. For articles presenting motor scores only in line graphs, we extracted the data from the figures using a graph digitizer software (Plot digitizer)^1^. We identified and excluded all possible duplicated patients by examining demographics, motor scores, and the authors’ affiliations.

### Statistical Analysis

Normal distribution of data was tested using the Shapiro–Wilk test. We compared the demographics and outcome measurements between the groups using independent *t*-tests, Mann–Whitney *U* tests, chi-square tests, or Fisher’s exact tests, as appropriate. Statistical significance was set at *p* ≤ 0.05. All analyses were performed using IBM SPSS statistics 25 (IBM Corp., Armonk, NY, USA).

## Results

### The Multi-country Cohort

From a total of 132 DYT1 patients who underwent DBS in our institutions, we collected the detailed clinical information of 18 DYT1 patients with reported suboptimal DBS responses. However, a thorough review of the case files led to an additional seven exclusions. Patients 12 and 13 had suboptimal responses to DBS as a result of suboptimal lead positions, and patient 14 had a secondary worsening of dystonia because of lead migration. These three patients revealed marked improvement following the surgical repositioning of the DBS leads. Patients 15, 16, and 17 had excellent responses to DBS (>80%) followed by worsening of dystonia; however, these patients had persistent improvement ≥30% at the last follow-up. Despite a marked improvement in the BFMDRS-M, functional improvement in patients 15 and 18 was compromised by skeletal deformities and action-induced muscle spasms, respectively. The clinical information of patients 12–18 is presented in Supplementary Table S1.

**Figure 1:**
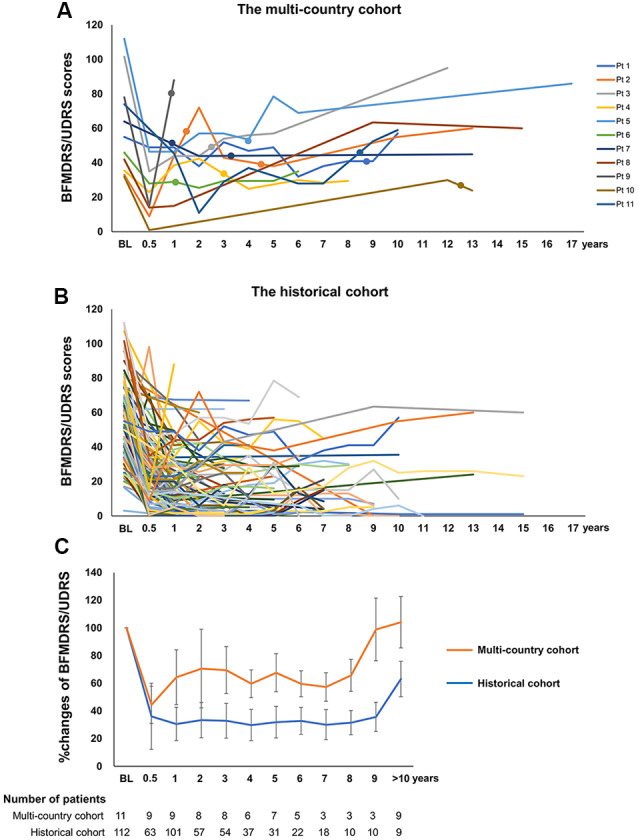
**(A)** Individual changes of the Burke-Fahn-Marsden Dystonia Rating Scale motor (BFMDRS-M) or Unified Dystonia Rating Scale (UDRS) in the multi-country cohort. **(B)** The closed circles on each line indicate additional GPi or subthalamic nucleus (STN) implantations. Individual changes of the BFMDRS-M or UDRS in the historical cohort. **(C)** Comparison of percent improvement of the BFMDRS-M/UDRS in the multi-country cohort and the historical cohort. Orange and blue line graphs represent mean scores for the multi-country cohort and the historical cohort, respectively. Whiskers represent standard errors. BFMDRS-M, Burke-Fahn-Marsden Dystonia Rating Scale motor score; UDRS, Unified Dystonia Rating Scale.

Compared to the preoperative baseline status, the remaining 11 patients (Table 1) had improvement that was less than 30% (range −81.8% to 29.7%) at the last clinical follow-up with a mean follow-up duration after implantation of 12.6 years (range 1.0–18.3 years). Nine patients had a family history of dystonia. The region of onset was the upper extremity in four patients and the lower extremity in seven with a mean age at onset of 6.9 years (range 5–10 years). In all patients, the dystonia became generalized within 2.5 years (mean 1.9 years, range 0.8–2.5 years), and the patients underwent DBS at a mean age of 12.5 years (range 8–23 years) with a mean disease duration before DBS of 5.6 years (range 2–17 years). All of the patients were initially treated with bilateral GPi DBS. Individual changes in the BFMDRS-M/UDRS are summarized in Figure 1A. Importantly, all patients showed motor improvement of ≥30% postoperatively (mean improvement of 59% at 6 months) but subsequently experienced secondary worsening of dystonia symptoms starting between 6 months and 3 years after DBS. Maximal motor improvement was observed between 6 months and 2 years with the improvement of 30–50% in four patients, 50–80% in four, and >80% in three. Cranial involvement was observed in six patients before surgery, and four patients developed cranial dystonia following surgery. Pseudo-dystonic orofacial movement or speech disorders due to the current spread into the surrounding structures were carefully ruled out by the participating expert DBS centers by use of empirical programming of the device and/or stopping the stimulation temporarily. The subscores, as well as the total scores of the BFMDRS/UDRS, were available for nine patients (Supplementary Table S2). In all the patients, the worsening of the subscores at long-term follow-ups was observed not only in the cranial regions but also in the limbs and trunk. The lead positions were measured at each expert center and judged optimal (Supplementary Table S3). Various stimulation settings were attempted through multiple programming sessions: i.e., single monopolar, double monopolar, or bipolar stimulation with combinations of different voltage, pulse width, and stimulation frequency. All patients underwent additional DBS surgeries targeting the GPi or subthalamic nucleus (STN). The timing of additional implantations for individual patients is shown in Figure 1A. Five patients underwent implantation of a second pair of leads within the bilateral GPi; three underwent additional bilateral STN implantation; one underwent replacement of bilateral GPi leads because of lead fractures; considering the asymmetric nature of dystonia symptoms, one underwent additional implantation within the unilateral STN, and one underwent additional implantation within the unilateral STN and GPi. There were no patients with meaningful benefits following additional implantations.

**Table 1:** Patient characteristics of the multi-country cohort.

Patients	Sex	Family history	Age at onset (years)	Region of onset	Body distribution before DBS							Disease duration before dystonia generalization (years)	Disease duration before DBS (years)	Age at DBS (years)	follow-up after DBS (years)	BFMDRS or UDRS			New regions after DBS	Additional DBS surgeries
					UF	LF	Lx	*N*	UE	LE	T					Baseline	Best	Last FU		
Pt 1	M	+	5	LE	-	+	-	+	+	+	+	2 years	6	11	9	55	32	57	UF	L STN
Pt 2	M	-	6	UE	-	-	+	-	+	+	+	2 years	2	8	12	33	9	60	UF, LF	R GPi, R STN
Pt 3	F	+	6	LE	-	-	+	-	+	+	+	2.5 years	3	9	12	101.5	48	95	LF	Bil GPi
Pt 4	M	+	8	UE	-	+	+	+	+	+	+	2 years	9	17	8	35.5	25	29.5	UF	Bil GPi
Pt 5	M	+	6	LE	+	+	-	+	+	+	+	2 years	17	23	17.5	112	46.5	86		Bil GPi
Pt 6	F	+	7	LE	-	+	+	+	+	+	+	1.5 years	8	15	6	46	28	35	UE	Bil GPi
Pt 7	M	+	10	UE	-	-	-	+	+	+	+	2.5 years	3	13	12.5	64	44	45	UF, LF, Lx	Bil GPi
Pt 8	F	+	9	LE	-	-	-	+	+	+	+	2 years	2	11	15	42	4.5	60	Lx	Replacement of Bil GPi
Pt 9	M	+	7	LE	-	-	-	+	+	+	+	9 months	2	9	1	78	15	88		Bil STN
Pt 10	F	-	6	UE	-	-	-	-	+	+	-	2 years	5	11	13	32	2	24	LF, Lx, N, T	Bil STN
Pt 11	M	+	6	LE	-	-	+	+	+	+	+	2 years	5	11	10	74	11	59	UF, LF	Bil STN

*Regions: UF, Upper face; LF, Lower face; Lx, Larynx; N, Neck; UE, Upper extremity; LE, Lower extremity; T, Trunck; Additional surgery: Bil, Bilateral; GPi, Globus pallidus interna; L, Left; R, Right; STN, Subthalamic nucleus; BFMDRS-M, Burke-Fahn-Marsden Dystonia Rating Scale motor score; DBS, deep brain stimulation; UDRS, Unified Dystonia Rating Scale*.

Despite these interventions, the improvement at the last follow-up was less than 30% as compared with the baseline status. When the stimulation was turned off, all the patients in the cohort experienced immediate worsening at the long-term follow-up.

### The Historical Cohort

**Figure 2:**
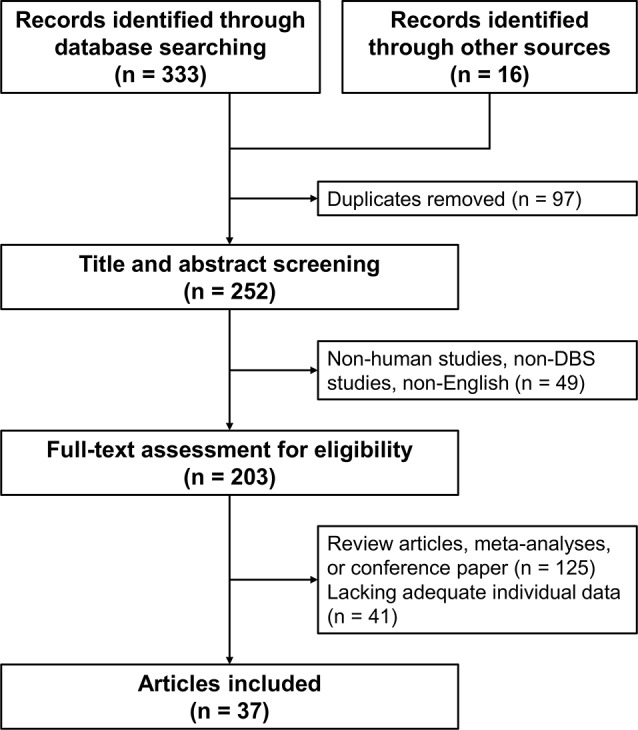
Flowchart of the literature search.

The flowchart of the systematic search and review process is shown in Figure 2. The list of 37 studies used for the historical cohort is presented in Supplementary Table S4. Twenty-four duplicated patients and five patients with reported suboptimal lead positions were removed. Consequently, a total of 112 DYT1 patients who met the inclusion criteria were included in the historical cohort. All the patients were treated with bilateral GPi DBS. Individual changes of BFMDRS-M/UDRS revealed sustained improvement of dystonia in most patients (Figure 1B). As shown in Figure 1C, the multi-country and historical cohorts showed distinct trajectories in the long-term. At the group level, the historical cohort experienced mean improvement of 64% at 6 months after surgery and had sustained improvement of approximately 65–70% up to 9 years; mean improvement at ≥10 years was less impressive (37%) because of worsening of scores in a subset of patients with a variable length of follow-up periods. Note that the historical cohort included a total of eight patients with suboptimal DBS responses from our earlier studies who were also included in the multi-country cohort (Cif et al., 2010; Krause et al., 2016; Tsuboi et al., 2019).

### Comparison Between the Multi-country Cohort and the Historical Cohort

We compared the historical cohort with the multi-country cohort who manifested suboptimal DBS responses for unexplained reasons (Table 2). Mean motor improvement at the last follow-up from baseline were 0% and 66% for the multi-country and historical cohorts, respectively. Compared with the historical cohort, the multi-country cohort had a significantly younger age at onset (*p* = 0.025), younger age at DBS (*p* = 0.019), and a significantly longer follow-up period after DBS (*p* < 0.001). Additionally, cranial involvement was more common in the multi-country cohort compared with the historical cohort (before DBS, 64% vs. 45%, *p* = 0.074; before or after DBS, 91% vs. 47%, *p* = 0.001).

**Table 2:** Comparison between the multi-country and historical cohorts.

	*n*	The multi-country cohort	*n*	The historical cohort	*p*
Age at onset (years)	11	7.0 ± 1.5	112	8.4 ± 2.8	0.025^a^
Age at DBS (years)	11	12.0 ± 5.0	112	18.6 ± 10.8	0.019^a^
Disease duration before DBS (years)	11	6.1 ± 4.9	112	10.2 ± 10.8	0.362
Gender (%male, male/female)	11	64%	101	56%	0.451
BFMDRS-M at baseline^b^	9	65.0 ± 28.8	107	61.2 ± 24.1	0.451
UDRS at baseline^b^	2	43.7 ± 11.0	5	44.0 ± 15.6	1.000
Cranial involvement before DBS	11	64%	76	45%	0.074
Cranial involvement before or after DBS	11	91%	76	47%	0.001^a^
Follow-up period after DBS (months)	11	10.5 ± 4.7	112	4.5 ± 4.0	<0.001^a^
%improvement of motor scales^c^	11	0% ± 35%	112	66% ± 33%	<0.001^a^

*Data are presented as mean ± SD unless otherwise indicated. ^a^*P* < 0.05 (significant). ^b^Motor assessments were done using either BFMDRS-M or UDRS. ^c^% improvement at the last follow-up compared with the preoperative scores. BFMDRS-M, Burke-Fahn-Marsden Dystonia Rating Scale motor score; DBS, deep brain stimulation; UDRS, Unified Dystonia Rating Scale*.

### Suboptimal and Good Responders in the Historical Cohort

In the historical cohort, 16 of 112 patients (14%) experienced suboptimal responses to DBS for unclear reasons at the last follow-up (<30% improvement). The clinical characteristics of the suboptimal and good responders are presented separately in Table 3. Individual data are shown in Supplementary Tables S5, S6, respectively. Mean motor improvement at the last follow-up from baseline was 4% and 77% for the suboptimal and good responders, respectively. Compared with the good responders, the suboptimal responders had a significantly younger age at onset (*p* = 0.005) and a significantly longer follow-up period after DBS (*p* = 0.020). Cranial involvement was more common in the suboptimal responders compared with the good responders (before DBS, 75% vs. 39%, *p* = 0.077; before or after DBS, 83% vs. 43%, *p* = 0.007).

**Table 3:** Comparison between the suboptimal and good responders of the historical cohort.

	*n*	Suboptimal responders	*n*	Good responders	*p*
Age at onset (years)	16	7.3 ± 2.0	96	8.6 ± 2.9	0.005^a^
Age at DBS (years)	16	21.1 ± 12.9	96	18.2 ± 10.5	0.645
Disease duration before DBS (years)	16	13.7 ± 12.0	96	9.6 ± 10.5	0.277
Gender (%male, male/female)	15	67%	86	55%	0.386
BFMDRS-M at baseline^b^	14	61.2 ± 24.1	93	51.7 ± 21.9	0.189
UDRS at baseline^b^	2	44.0 ± 15.6	3	46.7 ± 3.1	1.000
Cranial involvement before DBS	12	75%	64	39%	0.077
Cranial involvement before or after DBS	12	83%	64	43%	0.007^a^
Follow-up period after DBS (years)	16	7.3 ± 5.8	96	4.0 ± 3.4	0.020^a^
%improvement of motor scales^c^	16	4% ± 29%	96	77% ± 20%	<0.001^a^

*Data are presented as mean ± SD unless otherwise indicated. The two groups were compared using independent *t*-tests, Mann–Whitney U tests, chi-square tests, or Fisher’s exact tests. ^a^*P* < 0.05 (significant). ^b^Motor assessments were done using either BFMDRS-M or UDRS. ^c^% improvement at the last follow-up compared with the preoperative scores. BFMDRS-M, Burke-Fahn-Marsden Dystonia Rating Scale motor score; DBS, deep brain stimulation; UDRS, Unified Dystonia Rating Scale*.

The suboptimal responders can be divided into two groups (Table 4 and Supplementary Table S5): those with short disease duration before DBS (2–9 years) and those with very long disease duration before DBS (17.5–36 years). Both groups had similar age at onset (7.1 vs. 7.7 years old, *p* = 0.681) and similarly high rates of cranial involvement (both 75%, *p* = 0.745). However, the possible contribution of fixed skeletal deformities to suboptimal DBS response was described only in those with very long disease duration before DBS (*n* = 3).

**Table 4:** Comparison of the suboptimal responders based on disease duration before DBS.

	*n*	Patients with short disease duration before DBS	*n*	Patients with long disease duration before DBS	*p*
Age at onset (years)	9	7.1 ± 1.7	7	7.7 ± 2.4	0.681
Age at DBS (years)	9	11.4 ± 3.6	7	33.5 ± 9	<0.001^a^
Disease duration before DBS (years)	9	4.4 ± 2.8	7	25.8 ± 7.1	<0.001^a^
Gender (%male, male/female)	8	50%	7	86%	0.182
BFMDRS-M at baseline^b^	7	58.3 ± 23.3	7	64.1 ± 26.3	0.833
UDRS at baseline^b^	2	44.0 ± 15.6	0	NA	NA
%improvement of motor scales^c^	9	−5.8 ± 35.7	9	16.2 ± 9.4	0.252
Cranial involvement before DBS	8	75%	4	75%	0.745
Cranial involvement before or after DBS	8	88%	4	75%	0.576
Follow-up period after DBS (months)	9	107.3 ± 70.9	7	61.3 ± 63.5	0.338

*Data are presented as mean ± SD unless otherwise indicated. The two groups were compared using independent *t*-tests, Mann–Whitney U tests, chi-square tests, or Fisher’s exact tests. ^a^*P* < 0.05 (significant). ^b^Motor assessments were done using either BFMDRS-M or UDRS. ^c^% improvement at the last follow-up compared with the preoperative scores. BFMDRS-M, Burke-Fahn-Marsden Dystonia Rating Scale motor score; DBS, deep brain stimulation; NA, not available; UDRS, Unified Dystonia Rating Scale*.

## Discussion

The present study aimed to investigate the clinical characteristics of a multi-country cohort of patients with DYT1 dystonia who experienced suboptimal responses to DBS. Because only a few cases of DYT1 dystonia are operated each year even in expert centers, this dataset, though difficult to obtain, is crucial for the field. A few retrospective studies and a recent meta-analysis of mixed cohorts of inherited or idiopathic isolated dystonia patients reported that the *TOR1A* mutation-positive status was associated with a better DBS response, whereas the reasons underpinning suboptimal responses remained mostly unexplored (Vasques et al., 2009; Andrews et al., 2010; Borggraefe et al., 2010; FitzGerald et al., 2014; Moro et al., 2017). Our findings suggest that a subset of DYT1 patients may have a relatively more aggressive phenotype of DYT1 dystonia characterized by younger age at onset, faster disease progression before DBS, and cranial involvement. Patients with this phenotype may be more likely to experience suboptimal responses to DBS.

There is a surprising lack of published information on suboptimal responses to DBS in DYT1 dystonia despite a growing number of reported cases. Panov et al. (2013) reported that 47 DYT1 patients experienced an improvement in BFMDRS-M by greater than 80% up to 7 years after surgery; however, there was no description of patients with suboptimal DBS responses. Markun et al. (2012) observed improvement in BFMDRS-M by 70.3% in 14 DYT1 patients with a mean follow-up of 2.7 years and only commented on patients with skeletal deformities associated with suboptimal improvement. Cif et al. (2010) observed 59% improvement in 26 DYT1 patients with a mean follow-up of 6.2 years. Their cases showed less improvement compared with the former two cohorts; however, they included five cases (19%) who had suboptimal long-term DBS responses. Details of these (Cif et al., 2010) suboptimal patients are included as part of the multi-country cohort. Finally and importantly, most cases in the historical cohort also revealed sustained improvement in the long-term as contrasted to our multi-country cohort. The rate of suboptimal DBS responses due to unexplained reasons was approximately 8% (11 out of 132) in the multi-country cohort, which seems comparable to 14% in the historical cohort. Note that the data from the historical cohort should be interpreted with caution because of limited clinical information available on DBS programming and lead locations.

All 11 patients in our multi-country cohort initially experienced dystonia improvement of more than 30% postoperatively with subsequent worsening. Intriguingly, secondary worsening of dystonia symptoms began between 6 months and 3 years after DBS without any identifiable reasons, e.g., secondary skeletal deformities, cervical myelopathy, lead migrations, device malfunction, and suboptimal programming (Krauss et al., 2002; Anheim et al., 2008; Isaias et al., 2008; Picillo et al., 2016; Morishita et al., 2017; Pauls et al., 2017; Tsuboi et al., 2019). All the patients underwent additional DBS surgeries targeting the STN or GPi; however, these extra leads did not seem to provide a robust benefit. This finding is in agreement with the earlier study reporting variable responses to additional DBS implantations within DYT1 patients (Cif et al., 2012). Importantly, we observed a clear worsening of dystonia symptoms when DBS was turned off at the long-term follow-up visits, reinforcing the idea that DBS may still be a useful intervention even if the clinical outcomes are less robust. However, we cannot determine the relative contribution of disease progression and loss of benefits to secondary worsening because of the lack of formal on- and off-stimulation motor score comparisons (this was a limitation of the multi-country cohort and several cases treated worldwide). The involvement of body parts unaffected before surgery suggests disease progression likely played a role.

To the best of our knowledge, available studies have not analyzed the relationship between cranial involvement and DBS responses in DYT1 patients. Cranial involvement is less common in patients with DYT1 dystonia, with a range reported between 12.0% and 28.2% (Lee et al., 2012). However, cranial involvement in the multi-country cohort (before DBS, 64%; before or after DBS, 91%) was more frequently observed compared with the historical cohort and compared with the literature (Lee et al., 2012). Similarly, in the historical cohort, the suboptimal responders also showed a higher rate of cranial involvement as compared with the good responders. These findings strongly suggest the possible association between cranial involvement and suboptimal DBS responses in DYT1 patients.

The multi-country cohort had a young age at onset of dystonia ranging from 5 to 10 years old. The mean age at onset of the multi-country cohort was significantly younger when compared with the historical cohort (7.0 vs. 8.6 years old). The multi-country cohort required DBS treatment significantly younger than the historical cohort (mean, 12.0 vs. 19.0 years old) with relatively shorter disease duration (mean, 6.1 vs. 10.5 years, *p* > 0.05). Notably, dystonia evolved quickly to become generalized within 2.5 years after onset in all the patients of the multi-country cohort, and the motor symptoms progressed. Although the precise information on the time to a generalization of dystonia was not available for the historical cohort, the mean time to generalization of dystonia in DYT1 patients was previously reported at 3.1–8.4 (range, 1–30) years (Fasano et al., 2006; Lee et al., 2012), which was longer than that in the multi-country cohort. These results corroborate the multi-country cohort had faster disease progression with a younger age at onset.

Despite early intervention with DBS, the multi-country cohort experienced suboptimal outcomes in the long-term. This result appears to be contradictory to the earlier dystonia studies reporting the association between better outcomes and shorter disease duration before DBS (Isaias et al., 2011; Lumsden et al., 2013; Artusi et al., 2020). In the suboptimal responders included in the historical cohort, only those with very long disease duration before DBS were reported to have fixed skeletal deformities, and age at onset was similar between these two groups. Therefore, fixed skeletal deformities are thought to be responsible for their suboptimal DBS responses at least to some extent (Isaias et al., 2008; Pauls et al., 2017). Nevertheless, the multi-country cohort experienced secondary worsening following initial good responses to DBS without any identifiable reasons, and these patients were characterized by younger age at onset, faster disease progression before DBS, and cranial involvement. Because this phenotype of DYT1 dystonia is relatively less common (approximately 8%), analyses at the group level may not identify the presence of this phenotype. Thus, the present case series revealed a possible aggressive phenotype of DYT1 dystonia and provided detailed observations.

Although the genetics of DYT1 dystonia appear simple because virtually all cases have the same pathogenic deletion variant (c.907_909delGAG), the reduced penetrance and variable clinical phenomenology suggest that other genetic or environmental modifying factors influence phenotypic expression and that these factors may factor into responses to DBS or other treatments (Charlesworth et al., 2013; Weisheit et al., 2018). Dystonia patients carrying different variants in the *TOR1A* gene have been reported, but the consensus has yet to be reached regarding their pathogenicity (Martino et al., 2013; Siokas et al., 2019). A polymorphism in exon 4 of the DYT1 gene (rs1801968, D216H) was reported to affect the clinical penetrance of DYT1 (Risch et al., 2007). Additionally, the multi-country cohort was not tested for other dystonia-causing genes such as DYT-*THAP1* and DYT-*GNAL*, which are known to have a higher incidence of cranial involvement. Furthermore, some variants in brain-derived neurotrophic factor (BDNF) and apolipoprotein E (APOE) have also been reported to lead to an increased incidence of dystonia, possibly *via* altered neural plasticity (Siokas et al., 2019). These findings lead us to speculate on the intriguing possibility that variants within the *TOR1A* gene or other genes may affect the phenotypic manifestation of dystonia and the consequent DBS responses. The data from the current study cannot support or refute this hypothesis. Another unanswered question is the reason for secondary worsening after good initial responses to DBS. Curiously, secondary worsening of dystonia has also been reported in other genetic forms of dystonia, such as DYT-*THAP1* or *GNAO1* mutations (Panov et al., 2012; Brüggemann et al., 2015; Koy et al., 2018). Future neurophysiological and functional imaging studies will hopefully shed further light on the underlying pathophysiology.

There are several weaknesses in this work that should be considered. Because only a few cases of suboptimal responses to DBS are recorded even in expert centers, accumulating enough cases is challenging. Assembling this cohort required a multi-country effort. We would argue that collecting 11 patients with suboptimal outcomes provided valuable information. We could not analyze the whole cohort of the participating centers, which included patients with successful DBS outcomes. Because most studies included were observational unblinded studies, we did not perform a formal risk of bias assessments for the historical cohort. Although the findings from the historical cohort were similar to those from earlier single-center cohorts, the possibility of publication bias must be considered (Markun et al., 2012; Panov et al., 2013). Also, we had to exclude some articles because of insufficient individual clinical information. The historical cohort lacked the data of disease duration before dystonia generalization. The data from the historical cohort should be interpreted with caution because detailed information on DBS programming and lead locations were not available in most publications. The rate of suboptimal DBS responses in the historical cohort might increase with longer follow-up periods. The leads in the multi-country cohort were subjectively judged to be well-placed locally in each DBS center, based on expert opinions. We could not analyze the relationship between lead positions and DBS responses with a unified methodology because the digital imaging data were no longer available for some patients. Ideally, the accuracy of targeting within the posteroventrolateral GPi should be examined using advanced imaging analyses. Importantly, lead locations may potentially impact short-term effects as well as long-term therapeutic outcomes. The multi-country cohort attempted various stimulation settings through multiple programming sessions without a standardized programming protocol. Because the therapeutic effects of DBS in DYT1 patients are highly variable, we cannot exclude the possibility that there is room for improvement with higher stimulation intensity or different combinations of stimulation settings (Kupsch et al., 2011; Cif et al., 2013; Picillo et al., 2016). Finally, the threshold for a suboptimal response was chosen arbitrarily to exceed a placebo effect.

## Conclusion

Approximately 8% of patients represent a more aggressive phenotype of DYT1 dystonia characterized by younger age at onset, faster disease progression, and cranial involvement, which seems to be associated with suboptimal DBS responses in the long-term (e.g., secondary worsening). Importantly, the on-/off-stimulation comparison at the long-term follow-up demonstrated beneficial effects of DBS despite missing the 30% threshold for improvement over baseline. Therefore, DBS may still be a useful intervention, even if the clinical outcomes are less robust. Additional rescue STN or GPi DBS implantations may not provide meaningful improvement if the original leads were placed optimally. Patients with this phenotype may have different neuroplasticity, neurogenetics, or possibly distinct neurophysiology, although the exact differences underpinning DBS outcomes are unknown. Future studies should explore whether genetic variants within the *TOR1A* gene or other genes may determine DBS responses. This information could be useful for both clinicians and patients in clinical decision making and patient counseling before and following DBS implantations.

## Data Availability Statement

The datasets generated for this study are available on request to the corresponding author.

## Ethics Statement

The studies involving human participants were reviewed and approved by University of Florida Institutional Review Board. Written informed consent from the participants’ legal guardian/next of kin was not required to participate in this study in accordance with the national legislation and the institutional requirements.

## Author Contributions

TT: research project conception, organization, and execution; statistical analysis design and execution; manuscript writing of the first draft. LC, PC, JO, DR, YM, AL, PD, IH, FM, NS, LO, AW, and KF: research project execution; manuscript review and critique. JC: statistical analysis review and critique; manuscript review and critique. MO: research project conception, organization, and execution; manuscript review and critique.

## Conflict of Interest

The authors declare that the research was conducted in the absence of any commercial or financial relationships that could be construed as a potential conflict of interest.

## Footnotes

^1^http://plotdigitizer.sourceforge.net/
