# Supplementary material for: Secondary Worsening Following DYT1 Dystonia Deep Brain Stimulation: A Multi-country Cohort
Source: Front Hum Neurosci. 2020 Jun 25;14:242. doi: 10.3389/fnhum.2020.00242 (PMC7330126; doi:10.3389/fnhum.2020.00242)
Supplement: Supplementary file 1 [file Data_Sheet_1.docx]

Supplementary Material

Contents

- **Appendix 1.** Search syntax
- **Supplementary Tables 1–6.**
- **e-References**

Search syntax.

1) Pubmed

((((stimulation) OR neurostimulation) OR DBS)) AND ((DYT1) OR TOR1A)

2) Embase

(stimulation OR neurostimulation OR dbs) AND (dyt1 OR tor1a) AND [humans]/lim AND [english]/lim

3) Cochrane Library

#1 Stimulation

#2 Neurostimulation

#3 DBS

#4 TOR1A

#5 DYT1

#6 #1 OR #2 OR #3

#7 #4 OR #5

#8 #6 AND #7

| Supplementary Table 1. Clinical characteristics of the patients excluded. | | | | | | | | | | | | | | | | | | | | | |
| --- | --- | --- | --- | --- | --- | --- | --- | --- | --- | --- | --- | --- | --- | --- | --- | --- | --- | --- | --- | --- | --- |
| Patients† | Sex | Family history | Age at onset (years) | Region of onset | Body distribution before DBS | | | | | | | Disease duration before dystonia generalization (years) | Disease duration before DBS (years) | Age at DBS (years) | Follow-up after DBS (years) |  | BFMDRS or UDRS | | | New regions after DBS | Additional DBS surgeries |
|  |  |  |  |  | UF | LF | Lx | N | UE | LE | T |  |  |  |  |  | Baseline | Best | Last FU |  |  |
| Pt 12 | F | - | 8 | UE | - | - | - | + | + | + | + | 10 years | 24 | 32 | 18 |  | 45 | 7 | 7 |  | Repositioning of bil GPi |
| Pt 13 | M | + | 6 | UE | - | - | - | - | + | + | + | 6 months | 3 | 9 | 1 |  | 43 | 4.5 | 21.5 | N | Bil STN |
| Pt 14 | M | - | 6 | UE | - | - | + | + | + | + | + | 2 months | 2 | 8 | 12 |  | 71 | 7 | 36 | UF, LF | Bil STN |
| Pt 15 | F | + | 6 | LE | - | + | - | + | + | + | + | 4 years | 20 | 26 | 20 |  | 97.5 | 15 | 27 | UF | None |
| Pt 16 | M | + | 7 | UE | - | + | - | + | + | + | + | 9 months | 1.5 | 8.5 | 20 |  | 76 | 0 | 38 | UF | Bil GPi |
| Pt 17 | F | - | 6 | UE | - | - | - | - | + | + | + | 1.5 years | 2 | 8 | 5 |  | 12 | 2 | 8 | UE, LE | None |
| Pt 18 | M | - | 8 | LE | - | - | - | - | + | + | - | Segmental | 49 | 57 | 1 |  | 11 | - | 5 |  | None |
| Regions: UF = Upper face, LF = Lower face, Lx = Larynx, N = Neck, UE = Upper extremity, LE = Lower extremity, T = Trunck; Additional surgery: Bil = Bilateral, GPi = Globus pallidus interna, L = Left, R = Right, STN = Subthalamic nucleus; BFMDRS-M = Burke-Fahn-Marsden Dystonia Rating Scale motor score; DBS = deep brain stimulation; UDRS = Unified Dystonia Rating Scale. | | | | | | | | | | | | | | | | | | | | | |

| Supplementary Table 2. Changes of BFMDRS/UDRS total scores and subscores | | | | | | | | | | | | | | |
| --- | --- | --- | --- | --- | --- | --- | --- | --- | --- | --- | --- | --- | --- | --- |
|  | Baseline | | | |  | Best scores after DBS | | | |  | Long-term follow-ups | | | |
|  | Total scores | Face and larynx | Neck | Limbs and trunk |  | Total scores | Face and larynx | Neck | Limbs and trunk |  | Total scores | Face and larynx | Neck | Limbs and trunk |
| Pt 1 | 55 | 5 | 7 | 43 |  | 32 | 6 | 4 | 22 |  | 57 | 20 | 7 | 30 |
| Pt 2 | 33 | 3 | 0 | 30 |  | 9 | 1 | 0 | 8 |  | 55 | 19 | 0 | 36 |
| Pt 3 | 101.5 | 13.5 | 8 | 80 |  | 35 | 0 | 0 | 35 |  | 71.5 | 11.5 | 6 | 54 |
| Pt 4 | 35.5 | 3.5 | 8 | 24 |  | 23 | 6 | 4.5 | 12.5 |  | 44.5 | 14.9 | 4.5 | 25 |
| Pt 5 | 112 | 24 | 8 | 80 |  | 46.5 | 2 | 2 | 42.5 |  | 93 | 15 | 6 | 72 |
| Pt 6 | 46 | 2 | 3 | 41 |  | 26 | 1 | 0 | 25 |  | 50.5 | 3.5 | 2 | 45 |
| Pt 7 | 64 | 5 | 8 | 41 |  | 44 | 2.5 | 3 | 38.5 |  | 58.5 | 6 | 6 | 46.5 |
| Pt 10 | 32 | 0 | 0 | 32 |  | 2 | 0 | 0 | 2 |  | 24 | 4.5 | 1.5 | 18 |
| Pt 11 | 74 | 6 | 12 | 68 |  | 11 | 4 | 0 | 7 |  | 59 | 15 | 12 | 36 |
| The total scores with the subscores (face and larynx, neck, and limb and trunk) are shown. The data were not available for patient 8 and 9. BFMDRS-M = Burke-Fahn-Marsden Dystonia Rating Scale motor score; DBS = deep brain stimulation; UDRS = Unified Dystonia Rating Scale. | | | | | | | | | | | | | | |

| Supplementary Table 3. Lead positions for the multi-county cohort. | | |
| --- | --- | --- |
|  | Original electrodes (x, y, z) | Additional electrodes (x, y, z) |
| Pt 1^a^ | Left GPi: -20.9, 2.4, -5.3  Right GPi: 20.5, 0.3, -4.5 | Left STN: -10.9, -2.3, -5.9 |
| Pt 2^a^ | Left GPi: -18.9, 2.0, -2.3  Right GPi: 21.8, 4.0, -0.6 | Right GPi: 20.7, 6.9, -6.5  Right STN: 10.3, -0.3, -6.0 |
| Pt 4^b^ | Left GPi: 114.3, 109.9, 99.8  Right GPi: 81.3, 111.2, 99.7 | Left GPi: not available  Right GPi: not available |
| Pt 5^b^ | Left GPi: 117.4, 108.8, 100.0  Right GPi: 76.7, 108.2, 100.1 | Left GPi: not available  Right GPi: not available |
| Pt 6^b^ | Left GPi: 122.7, 89.6, 80.0  Right GPi: 82.0, 90.0, 81.5 | Left GPi: not available  Right GPi: not available |
| Pt 7^b^ | Left GPi: 116.4, 113.3, 93.3  Right GPi: 83,4, 112.1, 90.0 | Left GPi: not available  Right GPi: not available |
| Pt 8^a^ | Left GPi: no available  Right GPi: 15.5, 2.2, -5.0 | Left GPi: -19.3, 1.8, -5.6  Right GPi: 19.0, 2.8, -5.3 |
| Pt 9^a^ | Left GPi: -18.8, 2.8, -4.0  Right GPi: -18.6, 3.3, -4.3 | Left STN: not available  Right STN: not available |
| Pt 10^a^ | Left GPi: -20.9, 2.3, -8  Right GPi: 19.3, 1.8, -8.6 | Left STN: -11.5, 2.5, 4.0 |
| Pt 11^a^ | Left GPi: -21.0, 1.0, -4.4  Right GPi: 18.3, 2.9, -2.9 | Left STN: 10.5, -3.0, -4.4  Right STN: 12.0, -3.0, -8.0 |
| ^a^ The anatomical coordinates for the electrode tips relative to the midcommissural point are shown; x (mm), lateral to midline; y (mm), anterior to midcommissural point; z (mm), dorsal to intercommissural plane.  ^b^ The anatomical coordinates for the upper limit of the second deepest contact (electrode model 3389, Medtronic), which was confirmed to be aligned to the anatomical target. A direct targeting based on MRI with immediate post-operative control were used.  Electrode positions for patients 3 were not available. Abbreviations: GPi = globus pallidus internus, STN = subthalamic nucleus | | |

| Supplementary Table 4. The list of studies which reported individual demographic data and motor outcomes for DYT1 patients. | | |
| --- | --- | --- |
| Studies | Total Number of patients reported | Number of DYT1 patients who fulfilled the inclusion criteria |
| Alcindor et al. (2010)^e1^ | 1 | 1 |
| Anheim et al. (2008) ^e2^ | 2 | 2 |
| Ben-Haim et al. (2016) ^e3^ | 5 | 4 |
| Borggraefe et al. (2008) ^e4^ | 1 | 1 |
| Borggraefe et al. (2010) ^e5^ | 6 | 3 |
| Cersosimo et al. (2008) ^e6^ | 10 | 3 |
| Cheung et al. (2013) ^e7^ | 2 | 1 |
| Cif et al. (2010) ^e8^ | 26 | 26 |
| Dupre et al. (2018) ^e9^ | 1 | 1 |
| Eltahawy et al. (2004) ^e10^ | 15 | 0 |
| Goto et al. (2006) ^e11^ | 3 | 3 |
| Jin et al. (2012) ^e12^ | 1 | 1 |
| Krause et al. (2004) ^e13^ | 10 | 4 |
| Krause et al. (2016) ^e14^ | 8 | 6 |
| Lumsden et al. (2013) ^e15^ | 63 | 4 |
| Markun et al. (2012) ^e16^ | 14 | 13 |
| Mehrkens et al. (2009) ^e17^ | 18 | 3 |
| Mehrkens et al. (2010) ^e18^ | 5 | 3 |
| Miri et al. (2014) ^e19^ | 9 | 3 |
| Miyagi et al. (2013) ^e20^ | 2 | 2 |
| Park et al. (2016) ^e21^ | 36 | 4 |
| Parr et al. (2007) ^e22^ | 4 | 1 |
| Pauls et al. (2017) ^e23^ | 22 | 3 |
| Petrossian et al. (2013) ^e24^ | 14 | 3 |
| Schrader et al. (2011) ^e25^ | 6 | 1 |
| Sensi et al. (2009) ^e26^ | 11 | 1 |
| Sobstyl et al. (2008) ^e28^ | 1 | 1 |
| Sobstyl et al. (2011) ^e27^ | 1 | 1 |
| Starr et al. (2006) ^e30^ | 23 | 5 |
| Starr et al. (2014) ^e29^ | 6 | 5 |
| Tisch et al. (2007) ^e31^ | 15 | 0 |
| Tronnier et al. (2000) ^e32^ | 3 | 1 |
| Tsuboi et al. (2019) ^e33^ | 16 | 4 |
| Tustin et al. (2019) ^e34^ | 60 | 5 |
| Vasques et al. (2009) ^e35^ | 40 | 15 |
| Vidailhet et al. (2007) ^e36^ | 22 | 7 |
| Zorzi et al. (2005) ^e37^ | 12 | 1 |

| Supplementary Table 5. Suboptimal responders in the historical cohort. | | | | | | | | | | | |
| --- | --- | --- | --- | --- | --- | --- | --- | --- | --- | --- | --- |
| References | Sex | Age at onset (years) | Disease duration before DBS (years) | Age at DBS (years) | Follow-up after DBS (years) | Cranial involvement before DBS | Cranial involvement before or after DBS | BFMDRS or UDRS | | | |
|  |  |  |  |  |  |  |  | Baseline | Best | Last FU | Percentage improvement at last FU from baseline |
| e33 ^a^ | M | 5 | 6 | 11 | 108 | 1 | 1 | 55 | 32 | 57 | -4% |
| e33 ^a^ | M | 6 | 2 | 8 | 144 | 1 | 1 | 33 | 9 | 60 | -82% |
| e20 ^a^ | M | 7 | 2 | 9 | 12 | 0 | 0 | 78 | 15 | 88 | -13% |
| e20 ^a^ | F | 9 | 2 | 11 | 180 | 0 | 1 | 42 | 14 | 60 | -43% |
| e34 ^a^ | NA | 10 | 5.75 | 15.75 | 24 | NA | NA | 53 | 16 | 42 | 21% |
| e8 ^a^ | M | 8 | 9 | 17 | 96 | 1 | 1 | 35.5 | 23 | 29.5 | 17% |
| e8 ^a^ | F | 7 | 8 | 15 | 216 | 1 | 1 | 46 | 25.5 | 35 | 24% |
| e8 ^a^ | F | 6.5 | 2.75 | 9.25 | 144 | 1 | 1 | 101.5 | 35 | 95 | 6% |
| e14 ^a^ | F | 5 | 2 | 7 | 42 | 1 | 1 | 52 | 16 | 41 | 21% |
| e16 ^b^ | M | 5 | 22 | 27 | 28 | 1 | 1 | 74 | 60 | 60 | 19% |
| e8 ^b^ | M | 8 | 27.25 | 35.25 | 48 | 1 | 1 | 51 | 23 | 41 | 20% |
| e8 ^b^ | M | 6 | 17.5 | 23.5 | 204 | 1 | 1 | 112 | 46.5 | 86 | 23% |
| e36 ^b^ | F | 8 | 18 | 26 | 36 | NA | NA | 42 | 29 | 30 | 29% |
| e25 ^b^ | M | 12 | 36 | 48 | 26 | 0 | 0 | 33 | 29 | 29 | 12% |
| e23 ^b^ | M | 9 | 33 | 42 | 39 | NA | NA | 62 | 62 | 62 | 0% |
| e10 ^b^ | M | 6 | 26.7 | 32.7 | 48 | NA | NA | 75 | 67 | 67 | 11% |
| BFMDRS-M = Burke-Fahn-Marsden Dystonia Rating Scale motor score; DBS = deep brain stimulation; NA = not assessed; UDRS = Unified Dystonia Rating Scale.  ^a^ Patients with short disease duration before DBS in Table 4.  ^b^ Patients with long disease duration before DBS in Table 4. | | | | | | | | | | | |

| Supplementary Table 6. Good responders in the historical cohort. | | | | | | | | | | | |
| --- | --- | --- | --- | --- | --- | --- | --- | --- | --- | --- | --- |
| References | Sex | Age at onset (years) | Disease duration before DBS (years) | Age at DBS (years) | Follow-up after DBS (years) | Cranial involvement before DBS | Cranial involvement before or after DBS | BFMDRS or UDRS | | | |
|  |  |  |  |  |  |  |  | Baseline | Best | Last FU | Percentage improvement at last FU from baseline |
| e9 | M | 15 | 3 | 18 | 15 | 0 | 0 | 46 | 1 | 1 | 98 |
| e3 | F | 6 | 7 | 13 | 102 | NA | NA | 81 | 0 | 0 | 100 |
| e3 | M | 8 | 3 | 11 | 24 | 0 | 0 | 17 | 2 | 2 | 88 |
| e3 | M | 8 | 3 | 11 | 12 | 1 | 1 | 107 | 48 | 48 | 55 |
| e12 | F | 5 | 2 | 7 | 30 | 0 | 0 | 34 | 3 | 3 | 91 |
| e36 | F | 9 | 9 | 18 | 36 | NA | NA | 25 | 0 | 1 | 96 |
| e36 | M | 7 | 29 | 36 | 36 | NA | NA | 59.5 | 27.5 | 27.5 | 54 |
| e36 | M | 8 | 32 | 40 | 36 | NA | NA | 61 | 17 | 17 | 72 |
| e36 | M | 5 | 17 | 22 | 36 | NA | NA | 95.5 | 41 | 43 | 55 |
| e36 | F | 7 | 23 | 30 | 36 | NA | NA | 58.5 | 4.5 | 4.5 | 92 |
| e36 | M | 9 | 21 | 30 | 36 | NA | NA | 44.5 | 28 | 28 | 37 |
| e13 | M | 10 | 17.7 | 27.7 | 24 | NA | NA | 70 | 9.5 | 10 | 86 |
| e13 | M | 9 | 12.5 | 21.5 | 47 | NA | NA | 66.5 | 23.5 | 24.5 | 63 |
| e13 | M | 10 | 8.8 | 18.8 | 52 | NA | NA | 77 | 25 | 35 | 55 |
| e2 | NA | 10 | 38 | 48 | 12 | 0 | 0 | 82.5 | 37 | 37 | 55 |
| e2 | NA | 8 | 7 | 15 | 12 | 0 | 0 | 74.5 | 10 | 10 | 87 |
| e19 | F | 7 | 4 | 11 | 12 | NA | NA | 41 | 3 | 3 | 93 |
| e19 | F | 7 | 7 | 14 | 11 | NA | NA | 44 | 14 | 14 | 68 |
| e19 | F | 8 | 11 | 19 | 11 | NA | NA | 20 | 4 | 4 | 80 |
| e19 | M | 9 | 12 | 21 | 11 | NA | NA | 46 | 16 | 16 | 65 |
| e7 | M | 11 | 4 | 15 | 72 | 0 | 0 | 64 | 0 | 4 | 94 |
| e18 | M | 8 | 4 | 12 | 101 | 0 | 0 | 55 | 7 | 7 | 87 |
| e18 | F | 12 | 2 | 14 | 100 | 1 | 1 | 65 | 6 | 6 | 91 |
| e18 | F | 7 | 1 | 8 | 50 | 0 | 0 | 60 | 4 | 5 | 92 |
| e18 | F | 7 | 13 | 20 | 90 | NA | NA | 32 | 6 | 7 | 78 |
| e4 | F | 8 | 1 | 9 | 14 | 0 | 0 | 58 | 1 | 1 | 98 |
| e24 | M | 11 | 1.8 | 12 | 80 | 0 | 0 | 37 | 4 | 4 | 89 |
| e24 | M | 14 | 3 | 17 | 37 | 0 | 0 | 30 | 0 | 0 | 100 |
| e24 | M | 9 | 3 | 12 | 39 | 0 | 0 | 40 | 13 | 13 | 68 |
| e22 | M | 8 | 7 | 15 |  | 1 | 1 | 66 | 8 | 8 | 88 |
| e26 | F | 10 | 13 | 23 | 83 | 0 | 2 | 33 | 7 | 7 | 79 |
| e21 | M | 8 | 4 | 12 | 60 | NA | NA | 30 | 9.5 | 9.5 | 68 |
| e21 | M | 9 | 8 | 17 | 36 | NA | NA | 40.5 | 6 | 6 | 85 |
| e21 | M | 7 | 3 | 10 | 12 | NA | NA | 32.5 | 6.5 | 6.5 | 80 |
| e21 | M | 7 | 25 | 32 | 12 | NA | NA | 50 | 24 | 24 | 52 |
| e11 | M | 7 | 14 | 21 | 12 | 0 | 0 | 68.5 | 0 | 0 | 100 |
| e11 | M | 12 | 15 | 27 | 12 | 0 | 0 | 3 | 0.5 | 0.5 | 83 |
| e11 | M | 9 | 6 | 15 | 12 | 0 | 0 | 61 | 3 | 3 | 95 |
| e16 | M | 11 | 1 | 12 | 45 | 0 | 2 | 34 | 1 | 1 | 97 |
| e16 | F | 8 | 9 | 17 | 46 | 1 | 1 | 51.5 | 0 | 0 | 100 |
| e16 | M | 7 | 10 | 17 | 26 | 1 | 1 | 90 | 44 | 44 | 51 |
| e16 | M | 9 | 6 | 15 | 11 | 0 | 2 | 58 | 14 | 14 | 76 |
| e16 | M | 12 | 5 | 17 | 28 | 1 | 1 | 49.5 | 22 | 22.5 | 55 |
| e16 | F | 8 | 3 | 11 | 77 | 0 | 2 | 46 | 2 | 2 | 96 |
| e16 | M | 8 | 9 | 17 | 24 | 1 | 1 | 48.5 | 10 | 10 | 79 |
| e16 | F | 7 | 3 | 10 | 36 | 0 | 2 | 78 | 0 | 11.5 | 85 |
| e16 | F | 7 | 4 | 11 | 32 | 0 | 2 | 32 | 2 | 2 | 94 |
| e16 | F | 4 | 19 | 23 | 48 | 1 | 1 | 22 | 12 | 12 | 45 |
| e16 | M | 7 | 15 | 22 | 28 | 1 | 1 | 69.5 | 42 | 42 | 40 |
| e16 | F | 9 | 2 | 11 | 15 | 0 | 2 | 22 | 3 | 3 | 86 |
| e29 | M | 7 | 2 | 9 | 12 | 1 | 1 | 44 | 4 | 4 | 91 |
| e29 | M | 8 | 3 | 11 | 12 | 0 | 2 | 68 | 0 | 0 | 100 |
| e29 | M | 13 | 2 | 15 | 12 | 1 | 1 | 22.5 | 1 | 1 | 96 |
| e29 | F | 7 | 4 | 11 | 12 | 0 | 2 | 36.5 | 0 | 0 | 100 |
| e29 | M | 6 | 1 | 7 | 12 | 0 | 2 | 20 | 9 | 9 | 55 |
| e29 | NA | 8 | 9 | 17 | 15 | 0 | 2 | 52 | 0 | 0 | 100 |
| e29 | NA | 7 | 10 | 17 | 17 | NA | NA | 90 | 44 | 44 | 51 |
| e29 | NA | 9 | 6 | 15 | 14 | NA | NA | 58 | 14 | 14 | 76 |
| e29 | NA | 12 | 5 | 17 | 11 | 0 | 2 | 49.5 | 22 | 22 | 56 |
| e14 | M | 6 | 10 | 16 | 119 | NA | NA | 74.5 | 34 | 35.5 | 52 |
| e14 | F | 7 | 5 | 12 | 156 | NA | NA | 41 | 9 | 24 | 41 |
| e14 | M | 7 | 1 | 8 | 20 | 1 | 1 | 50 | 1.5 | 1.5 | 97 |
| e14 | F | 10 | 7 | 17 | 20 | NA | NA | 36 | 17 | 20 | 44 |
| e6 | F | 6 | 4 | 10 | 93 | NA | NA | 67 | 34 | 45 | 33 |
| e6 | M | 24 | 4 | 28 | 35 | NA | NA | 16.5 | 4 | 5 | 70 |
| e6 | F | 5 | 4 | 9 | 33 | NA | NA | 45 | 21 | 21 | 53 |
| e27 | F | 14 | 3 | 17 | 24 | 0 | 0 | 25 | 5 | 5 | 80 |
| e28 | M | 7 | 17 | 24 | 60 | 0 | 0 | 112 | 11 | 23 | 79 |
| e34 | NA | 6.5 | 0.8 | 7.3 | 24 | NA | NA | 50 | 6 | 22.5 | 55 |
| e34 | NA | 10.25 | 3.65 | 13.9 | 24 | NA | NA | 57 | 9 | 9 | 84 |
| e34 | NA | 9 | 4.75 | 13.75 | 12 | NA | NA | 25 | 10 | 10 | 60 |
| e34 | NA | 8 | 3.75 | 11.75 | 24 | NA | NA | 59.5 | 10 | 12.5 | 79 |
| e8 | F | 7.5 | 3 | 10.5 | 120 | 1 | 1 | 81 | 0 | 0 | 100 |
| e8 | F | 6 | 20 | 26 | 120 | 1 | 1 | 97.5 | 10 | 10 | 90 |
| e8 | F | 7 | 7 | 14 | 108 | 1 | 1 | 63 | 0 | 5 | 92 |
| e8 | M | 7 | 1.5 | 8.5 | 240 | 0 | 1 | 76 | 0 | 38 | 50 |
| e8 | M | 9 | 4 | 13 | 90 | 0 | 0 | 51 | 0 | 15 | 71 |
| e8 | F | 11.5 | 2 | 13.5 | 90 | 0 | 0 | 24 | 0 | 15.5 | 35 |
| e8 | F | 9 | 4.5 | 13.5 | 90 | 1 | 1 | 37.5 | 4 | 21 | 44 |
| e8 | M | 8 | 5 | 13 | 84 | 0 | 0 | 37 | 0 | 4 | 89 |
| e8 | M | 7 | 6 | 13 | 84 | 1 | 1 | 65.5 | 6 | 8.5 | 87 |
| e8 | M | 6.5 | 35 | 41.5 | 84 | 1 | 1 | 84.5 | 28.5 | 29 | 66 |
| e8 | M | 6 | 7 | 13 | 72 | 1 | 1 | 69 | 0 | 2 | 97 |
| e8 | F | 7 | 1.5 | 8.5 | 72 | 0 | 0 | 35.5 | 0 | 0 | 100 |
| e8 | F | 7 | 43 | 50 | 66 | 1 | 1 | 28 | 1 | 4.5 | 84 |
| e8 | F | 20 | 5 | 25 | 66 | 0 | 0 | 38 | 0 | 2 | 95 |
| e8 | F | 8 | 28.5 | 36.5 | 63 | 1 | 1 | 67 | 10.5 | 16 | 76 |
| e8 | F | 6.5 | 12.5 | 19 | 60 | 0 | 0 | 64 | 0 | 0 | 100 |
| e8 | F | 9 | 56.5 | 65.5 | 51 | 0 | 0 | 27.5 | 10.5 | 10.5 | 62 |
| e8 | M | 8 | 40 | 48 | 48 | 1 | 1 | 61.5 | 21 | 25 | 59 |
| e8 | F | 9 | 3 | 12 | 45 | 0 | 0 | 40 | 0 | 5 | 88 |
| e8 | F | 6 | 3 | 9 | 42 | 0 | 0 | 42 | 3 | 3 | 93 |
| e8 | F | 9 | 1.5 | 10.5 | 36 | 1 | 1 | 60 | 0 | 0 | 100 |
| e33 | F | 12 | 12 | 24 | 156 | 1 | 1 | 50 | 6 | 23 | 54 |
| e33 | M | 8 | 24 | 32 | 108 | 1 | 1 | 44 | 0 | 0 | 100 |
| e15 | M | 11.2 | 3 | 14.2 | 12 | NA | NA | 24 | 5 | 5 | 79 |
| BFMDRS-M = Burke-Fahn-Marsden Dystonia Rating Scale motor score; DBS = deep brain stimulation; NA = not assessed; UDRS = Unified Dystonia Rating Scale. | | | | | | | | | | | |

**e-References**

e1. Alcindor D, Oh MY, Baser S, Angle C, Cheng BC, Whiting D: Stimulation of the globus pallidus internus in a patient with DYT1-positive primary generalized dystonia: a 10-year follow-up. Neurosurg Focus 29:E16, 2010

e2. Anheim M, Vercueil L, Fraix V, Chabardès S, Seigneuret E, Krack P, et al: Early stimulation of DYT1 primary generalized dystonia prevents from its secondary irreversible complications. Mov disord 23:2261–3, 2008

e3. Ben-Haim S, Flatow V, Cheung T, Cho C, Tagliati M, Alterman RL: Deep Brain Stimulation for Status Dystonicus: A Case Series and Review of the Literature. Stereotact Funct Neurosurg 94:207–5, 2016

e4. Borggraefe I, Boetzel K, Boehmer J, Berweck S, Mueller-Felber W, Mueller K, et al: Return to participation - significant improvement after bilateral pallidal stimulation in rapidly progressive DYT-1 dystonia. Neuropediatrics 39:239–42, 2008

e5. Borggraefe I, Mehrkens JH, Telegravciska M, Berweck S, Bötzel K, Heinen F: Bilateral pallidal stimulation in children and adolescents with primary generalized dystonia--report of six patients and literature-based analysis of predictive outcomes variables. Brain Dev 32:223–8, 2010

e6. Cersosimo MG, Raina GB, Piedimonte F, Antico J, Graff P, Micheli FE: Pallidal surgery for the treatment of primary generalized dystonia: long-term follow-up. Clin Neurol Neurosurg 110:145–50, 2008

e7. Cheung T, Zhang C, Rudolph J, Alterman RL, Tagliati M: Sustained relief of generalized dystonia despite prolonged interruption of deep brain stimulation. Mov disord 28:1431–4, 2013

e8. Cif L, Vasques X, Gonzalez V, Ravel P, Biolsi B, Collod-Beroud G, et al: Long-term follow-up of DYT1 dystonia patients treated by deep brain stimulation: an open-label study. Mov disord 25:289–99, 2010

e9. Dupre DA, Nangunoori R, Koduri S, Angle C, Cantella D, Whiting D: Disease Stabilization of DYT1-Positive Primary Generalized Dystonia With Deep Brain Stimulation of the Globus Pallidus Interna: A 15-Year Follow-up. Oper Neurosurg 14:597, 2018

e10. Eltahawy HA, Saint-Cyr J, Giladi N, Lang AE, Lozano AM: Primary dystonia is more responsive than secondary dystonia to pallidal interventions: outcome after pallidotomy or pallidal deep brain stimulation. Neurosurgery 54:613–19; discussion 619-21, 2004

e11. Goto S, Yamada K, Shimazu H, Murase N, Matsuzaki K, Tamura T, et al: Impact of bilateral pallidal stimulation on DYT1-generalized dystonia in Japanese patients. Mov disord 21:1785–7, 2006

e12. Jin ST, Lee MK, Ghang JY, Jeon SM: Deep Brain Stimulation of the Globus Pallidus in a 7-Year-Old Girl with DYT1 Generalized Dystonia. J Korean Neurosurg Soc 52:261–3, 2012

e13. Krause M, Fogel W, Kloss M, Rasche D, Volkmann J, Tronnier V: Pallidal stimulation for dystonia. Neurosurgery 55:1361–8; discussion 1368-70, 2004

e14. Krause P, Lauritsch K, Lipp A, Horn A, Weschke B, Kupsch A, et al: Long-term results of deep brain stimulation in a cohort of eight children with isolated dystonia. J Neurol 263:2319–26, 2016

e15. Lumsden DE, Kaminska M, Gimeno H, Tustin K, Baker L, Perides S, et al: Proportion of life lived with dystonia inversely correlates with response to pallidal deep brain stimulation in both primary and secondary childhood dystonia. Dev Med Child Neurol 55:567–74, 2013

e16. Markun LC, Starr PA, Air EL, Marks WJ, Volz MM, Ostrem JL: Shorter disease duration correlates with improved long-term deep brain stimulation outcomes in young-onset DYT1 dystonia. Neurosurgery 71:325–30, 2012

e17. Mehrkens JH, Bötzel K, Steude U, Zeitler K, Schnitzler A, Sturm V, et al: Long-term efficacy and safety of chronic globus pallidus internus stimulation in different types of primary dystonia. Stereotact Funct Neurosurg 87:8–17, 2009

e18. Mehrkens JH, Borggraefe I, Feddersen B, Heinen F, Bötzel K: Early globus pallidus internus stimulation in pediatric patients with generalized primary dystonia: long-term efficacy and safety. J Child Neurol 25:1355–61, 2010

e19. Miri S, Ghoreyshi E, Shahidi GA, Parvaresh M, Rohani M, Saffari M: Deep brain stimulation of globus pallidus internus for DYT1 positive primary generalized dystonia. Med J Islam Repub Iran 28:18, 2014

e20. Miyagi Y, Koike Y: Tolerance of early pallidal stimulation in pediatric generalized dystonia. J Neurosurg Pediatr 12:476–82, 2013

e21. Park HR, Lee JM, Ehm G, Yang H-J, Song IH, Lim YH, et al: Long-Term Clinical Outcome of Internal Globus Pallidus Deep Brain Stimulation for Dystonia. PLoS One 11:e0146644, 2016

e22. Parr JR, Green AL, Joint C, Andrew M, Gregory RP, Scott RB, et al: Deep brain stimulation in childhood: an effective treatment for early onset idiopathic generalised dystonia. Arch Dis Child 92:708–11, 2007

e23. Pauls KAM, Krauss JK, Kämpfer CE, Kühn AA, Schrader C, Südmeyer M, et al: Causes of failure of pallidal deep brain stimulation in cases with pre-operative diagnosis of isolated dystonia. Parkinsonism Relat Disord 43:38–48, 2017

e24. Petrossian MT, Paul LR, Multhaupt-Buell TJ, Eckhardt C, Hayes MT, Duhaime A-C, et al: Pallidal deep brain stimulation for dystonia: a case series. J Neurosurg Pediatr 12:582–7, 2013

e25. Schrader C, Capelle H-H, Kinfe TM, Blahak C, Bäzner H, Lütjens G, et al: GPi-DBS may induce a hypokinetic gait disorder with freezing of gait in patients with dystonia. Neurology 77:483–8, 2011

e26. Sensi M, Cavallo MA, Quatrale R, Sarubbo S, Biguzzi S, Lettieri C, et al: Pallidal stimulation for segmental dystonia: long term follow up of 11 consecutive patients. Mov disord 24:1829–35, 2009

e27. Sobstyl M, Ząbek M, Dzierzęcki S, Mossakowski Z, Szczałuba K: Successful bilateral pallidal stimulation in a patient with isolated lower limb dystonia coexistent with Langerhans cell histiocytosis and coeliac disease. Neurol Neurochir Pol 45:514–9, 2011

e28. Sobstyl M, Zabek M, Koziara H, Dzierczeki S: Chronic bilateral pallidal stimulation in a patient with DYT-1 positive primary generalized dystonia. A long-term follow-up study. Neurol Neurochir Pol 42:50–54, 2008

e29. Starr PA, Markun LC, Larson PS, Volz MM, Martin AJ, Ostrem JL: Interventional MRI-guided deep brain stimulation in pediatric dystonia: first experience with the ClearPoint system. J Neurosurg Pediatr 14:400–8, 2014

e30. Starr PA, Turner RS, Rau G, Lindsey N, Heath S, Volz M, et al: Microelectrode-guided implantation of deep brain stimulators into the globus pallidus internus for dystonia: techniques, electrode locations, and outcomes. J Neurosurg 104:488–501, 2006

e31. Tisch S, Zrinzo L, Limousin P, Bhatia KP, Quinn N, Ashkan K, et al: Effect of electrode contact location on clinical efficacy of pallidal deep brain stimulation in primary generalised dystonia. J Neurol Neurosurg Psychiatry 78:1314–9, 2007

e32. Tronnier VM, Fogel W: Pallidal stimulation for generalized dystonia. Report of three cases. J Neurosurg 92:453–6, 2000

e33. Tsuboi T, Jabarkheel Z, Foote KD, Okun MS, Wagle Shukla A: Importance of the initial response to GPi deep brain stimulation in dystonia: A nine year quality of life study. Parkinsonism Relat Disord 64:249–55, 2019

e34. Tustin K, Elze MC, Lumsden DE, Gimeno H, Kaminska M, Lin J-P: Gross motor function outcomes following deep brain stimulation for childhood-onset dystonia: A descriptive report. Eur J Paediatr Neurol 23:473–83, 2019

e35. Vasques X, Cif L, Gonzalez V, Nicholson C, Coubes P: Factors predicting improvement in primary generalized dystonia treated by pallidal deep brain stimulation. Mov disord 24:846–53, 2009

e36. Vidailhet M, Vercueil L, Houeto JL, Krystkowiak P, Lagrange C, Yelnik J, et al: Bilateral, pallidal, deep-brain stimulation in primary generalised dystonia: a prospective 3 year follow-up study. Lancet Neurol 6:223–9, 2007

e37. Zorzi G, Marras C, Nardocci N, Franzini A, Chiapparini L, Maccagnano E, et al: Stimulation of the globus pallidus internus for childhood-onset dystonia. Mov disord 20:1194–200, 2005
